# A systematic review of the measurement of the renin-angiotensin and kallikrein-kinin system peptides in normotension and hypertension

**DOI:** 10.1038/s41371-026-01186-x

**Published:** 2026-07-30

**Authors:** Elliot J. Gyedu, Sian Jenkins, Dan Lane, Ammar Akram, Farah Salim, Leong L. Ng, Donald J.L. Jones, Dennis Bernieh, Pankaj Gupta

**Affiliations:** 1 Department of Cardiovascular Sciences, University of Leicester, Leicester, UK; 2 Leicester van Geest MultiOMICS Facility, University of Leicester, Leicester, UK; 3 Department of Psychology and Vision Sciences, University of Leicester, Leicester, UK

**Keywords:** Hypertension, Risk factors

## Abstract

Hypertension is the leading modifiable risk factor for cardiovascular diseases and affects over 1.3 billion adults. The renin-angiotensin and kallikrein-kinin systems (RAS and KKS) are critical in regulating blood pressure (BP). Clinically, the RAS and KKS have gained renewed importance, especially due to the discovery of the non-canonical vasodilative RAS pathway. Thus, for their clinical use, clarity on their measurement and reference ranges is essential. This systematic review aimed to synthesise the reported baseline circulating concentrations of RAS and KKS peptides, and the pre-analytical and analytical methods used in measuing them, in normotensive and untreated hypertensive adults. It adhered to PRISMA guidelines and synthesised data from 27 of 2864 retrieved studies that met the inclusion criteria. Reported peptide concentrations varied widely, ranging from 0.06–81.85 × 10^3^ fmol/mL. However, there was substantial heterogeneity across included studies in sample collection procedures, protease inhibitor use for peptide stabilisation, participant clinical phenotyping, BP reporting, and analytical methods. Reported levels of several RAS and KKS peptides were generally lower in hypertensive cohorts than in normotensive cohorts; however, these differences should be interpreted cautiously given the small number of hypertensive studies, incomplete BP reporting, and substantial heterogeneity across all included studies. Current evidence is insufficient to establish reference ranges for RAS and KKS peptides. This review emphasises the need for standardisation of sampling protocols, comprehensive peptide stabilisation, consistent clinical phenotyping, and robust analytical methods before RAS and KKS peptides can be used as clinical markers and their therapeutic potential identified.

## Introduction

Hypertension is the most important modifiable risk factor for the prevention of cardiovascular morbidity and mortality [1]. According to the World Health Organisation (WHO), more than 1.3 billion adults worldwide have hypertension, and less than 50% have their blood pressure (BP) under control despite the availability of multiple antihypertensive therapies [1–3].

**Fig. 1:**
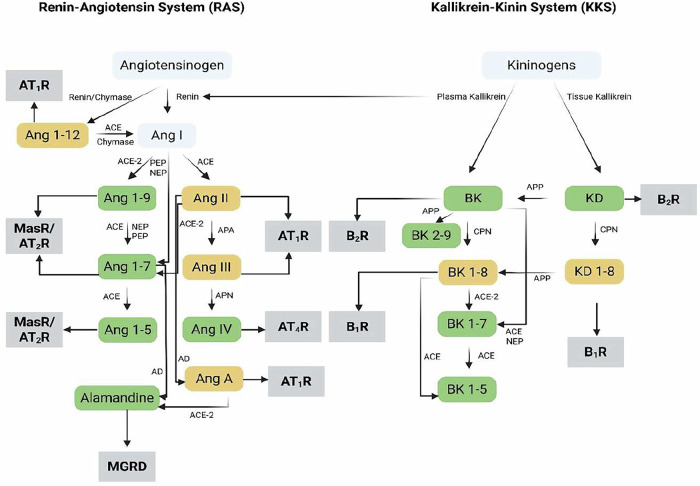
Overview of the Renin-Angiotensin System (RAS) and Kallikrein-Kinin System (KKS). The crosstalk between both systems is evident with enzymes leading to the formation of the peptides present in both and the plasma kallikrein enzyme activating both KKS and RAS. Precursors are in light blue, vasodilative peptides are in green, vasoconstrictive peptides in orange, and receptors in grey. Table key: ACE – Angiotensin-Converting Enzyme, ACE-2 – Angiotensin-Converting Enzyme-2, AD - Aspartate Decarboxylase, AGT - Angiotensinogen, Ang - Angiotensin, APA - Aminopeptidase A, APN - Aminopeptidase N, APP - Aminopeptidase P, AT_1_R- Angiotensin Type 1 Receptor, AT_2_R – Angiotensin Type 2 Receptor, BK – Bradykinin, CPN – Carboxypeptidase, MGRD - MAS-related G-protein-coupled receptor, NEP - Neprilysin, PEP - Prolylendopeptidase. Created with BioRender.com.

The renin-angiotensin system (RAS) (Fig. 1) is central to BP regulation. It is a complex network of peptides, enzymes, and receptors, which act in tandem to maintain vascular tone, fluid balance and electrolyte homeostasis [4, 5]. The canonical RAS pathway includes peptides such as Angiotensin III (Ang III), Angiotensin II (Ang II), and Angiotensin 1–12 (Ang 1–12), which contribute to vasoconstriction, inflammation and vascular remodelling [6, 7] through angiotensin II type 1 receptor (AT_1_R) [5, 8].

In contrast, the non-canonical RAS pathway includes peptides such as Angiotensin 1–7 (Ang 1–7) and Alamandine, which are associated with vasodilation, natriuresis, and anti-inflammatory effects through the Mas receptor (MasR) and Mas-related G-protein-coupled receptor D (MRGD) respectively [5, 6].

The kallikrein-kinin system (KKS) (Fig. 1), which interacts with the RAS, also contributes to BP regulation, vasodilation, and fluid and electrolyte homeostasis [6]. Its major peptides, bradykinin (BK) and kallidin (KD), together with their degradative products exert these vasoactive effects through the G-protein-coupled bradykinin type 1 receptor (B_1_R) or bradykinin type 2 receptor (B_2_R) [9, 10].

The RAS and KKS are also clinically relevant because many antihypertensive treatments, such as angiotensin converting enzyme inhibitors (ACEi) and angiotensin receptor blockers (ARBs), directly influence levels of RAS and KKS peptides through their mechanism of action [11, 12].

Furthermore, newer therapies including sodium-glucose co-transporter-2 (SGLT2) inhibitors, small interfering RNA (siRNA) agents and aldosterone synthase inhibitors [11–15] may also have an inadvertent effect on these pathways, limiting the vasodilative potential of non-canonical RAS and the KKS.

Thus, it is important that reference ranges for RAS and KKS peptides are developed and used in the clinical and research context to determine their association with clinical phenotypes and treatment therapies.

However, the biological and clinical interpretation of circulating RAS and KKS peptides remains challenging. This is due to variability during sample collection such as postural position, sample type, inclusion of protease inhibitors, and sample analysis techniques such as immunoassays and mass spectrometry (MS); all of which influence measured circulating peptide levels [6, 16–18].

These methodological issues are especially important because several RAS and KKS peptides are rapidly degraded ex vivo and differ by only a few amino acids [6], making accurate quantification difficult.

This systematic review aimed to synthesise the reported baseline circulating concentrations of RAS and KKS peptides, and the pre-analytical and analytical methods used to measure them, in normotensive and untreated hypertensive adults.

## Methods

### Eligibility criteria

The review was pre-registered on PROSPERO (ID: CRD42024524828) and used the PRISMA 2020 reporting guidelines for systematic reviews [19]. Studies were included if after title and abstract screening, the full text detailed measurement of a RAS or KKS peptide, consisted of adult participants aged 18 and above, who were healthy with normotension or diagnosed with hypertension (defined as clinical reading of systolic BP (SBP) ≥ 140 and/or diastolic BP (DBP) ≥ 90 mmHg, home BP readings of SBP ≥ 135 and/or DBP ≥ 85 mmHg or ambulatory BP readings of 24 hr average of SBP ≥ 130 and/or DBP ≥ 80 mmHg), with discontinued anti-hypertensive medications and washout period. Observational studies, cohort studies, and randomised controlled trials (RCTs) with baseline data, published in English, were included. Grey literature, including conference abstracts were excluded as this review required detailed information on sample collection procedures, peptide stabilisation strategies, and analytical methodologies, which are incompletely reported in non-full text.

### Study selection

Electronic databases including MEDLINE, Scopus, Web of Science, and Cochrane Central Register of Trials were searched from inception till July 22^nd^, 2025. The search strategy followed the PICO criteria [20], and be viewed in the Supplementary Material. ProQuest RefWorks Citation Manager 2024 was used to store retrieved papers and remove duplicates. Abstracts were reviewed by one reviewer (E.G.) in the first stage screening, with a random 10% screened by a second reviewer (S.J.). Disagreements were resolved after discussion between the two reviewers. The second stage screening reviewed the full text of articles and was completed by one reviewer (E.G.).

### Data extraction

Extracted data were retrieved and collated onto a customised data extraction form using Microsoft Excel 2023. Details collected included participant characteristics, type of study and study procedure, type of sample preparation and analysis methodology, measurement of peptides, and conclusions. Please refer to Tables 1 and 2 for the list of relevant variables and outcomes reported. The effect measure for all main outcomes was mean ( ± standard deviation).

**Table 1:** Study Characteristics for Normotensive Studies.

Author	Sample Size	Gender (M/F)^#^	Age (yr) (Mean ± S.D)^#^	Ethnicity	Index Test	BP Readings		Protease Inhibitor Used	Sample Analysis Methods	Peptides Measured
						SBP ( ± S.D)^#^	DBP( ± S.D)^#^			
Cohall et al. [26]	51	25/26	36.0 ± 9.0	Afro-Caribbean	24 hr ABPM	121.7 ± 13.4 (M) 120.5 ± 15.0 (F)	75.7 ± 7.9 (M) 75.0 ± 8.6 (F)	0.44 mM 1,20 ortho-phenanthroline monohydrate, 0.12 mM pepstatin and 1 mM Na p-hydroxymercuribenzoate	Radioimmunoassay	Ang II, Ang 1–7
Ferrario et al. [49]	52	30/22	54.5 ± 8.5	**-**	CBP	117.0 ± 13.0 (M) 117.0 ± 14.0 (F)	74.0 ± 8.0 (M) 72.0 ± 13.0 (F)	1,10-ortho-phenanthroline monohydrate (0.5 mmol/L); PHMB	Radioimmunoassay	Ang 1–12
Gafane-Matemane et al. [28]	1116	527/589	24.5 ± 3.2 (M) 24.6 ± 3.1 (F)	Black and White South Africans	CBP	117 ± 11.9 (M) 116 ± 12.5 (F)	79.6 ± 8.4 (M) 77.8 ± 8.1 (F)	Not mentioned	LC-MS/MS	Ang I, Ang II
Merrill et al. [36]	15	0/15	-	-	CBP	-	-	EDTA, ortho-phenantroline pepstatin and PCMB	Radioimmunoassay	Ang I, Ang II, Ang 1–7
Jankowski et al. [53]	7	4/3	63.0 ± 7.0	-	CBP	117.0 ± 5.0	66.0 ± 3.0	Not mentioned	MALDI-TOF	Ang A
Nogueira et al. [37]	12	0/12	-	-	CBP	**-**	-	PHMB, o-phenantroline, para-methylsulfonyl fluoride, pepstatin A and EDTA	Radioimmunoassay	Ang I, Ang II, Ang 1–7
Reyes-Engel et al. [38]	93	43/50	-	-	CBP	126.0 ± 7.0	76.0 ± 5.0	EDTA, o-phenanthrolene, PCMB, pepstatin A).	Radioimmunoassay	Ang I, Ang II, Ang 1–7
Shen et al. [30]	22	-	-	-	-	-	-	Not mentioned	LC-MS/MS	Ang I, Ang II, Ang III, Ang IV, Ang 1–5, Ang 1–7, Ang 1–9, Ang 1–12, BK
Silva et al. [39]	33	16/17	51.0 ± 18.0	-	-	-	-	0.44 mmol/ L o-phenanthroline, 1 mmol/L Na+parachloromercuribenzoate and 25 mmol/L EDTA	Radioimmunoassay	Ang II, Ang 1–7
Ayada et al. [29]	27	-	-	-	-	-	-	Not mentioned	ELISA	Ang I, Ang II, Ang 1–7
Braz et al. [58]	30	0/30	46.3 ± 7.7	-	--	-	-	Not mentioned	ELISA	Ang II, Ang 1–7
De Barros et al. [44]	24	11/14	39.6 ± 11.6	-	-	-	-	Not mentioned	ELISA	Ang II, Ang 1–7
Dorr et al. [51]	16	-	-	-	-	-	-	Not mentioned	LC-MS/MS	Ang II, Ang 1–7
Kovarik et al. [23]	10	-	-	-	-	-	-	EDTA, 1,10-phenanthroline, pepstatin A, p-PHMB, AEBSF, and specific inhibitors for renin and aminopeptidases A and N	LC-MS/MS	Ang I, Ang 2–10, Ang 1–8, Ang 2–8, Ang 1–5, Ang 1–7, Ang 1–9, Ang 3–8, Ang 2–7, Ang 3–7
Wolf et al. [27]	8	3/5	52.0 ± 21.0	-	CBP	135.0	84.0	2 mM AEBSF, 0.3 µM aprotinin, 130 µM bestatin, 1 mM EDTA, 14 µM E-64 and 1 µM leupeptin	LC-MS/MS	Ang I, Ang II, Ang III, Ang IV, Ang 1–5, Ang 1–7
Yang et al. [46]	50	27/23	64.0 ± 13.2	-	CBP	-	-	Not mentioned	Fluorimetry	Ang II, Ang 1–7
Alfaro et al. [8]	27	18/9	51.0 ± 14.0	-	-	-	-	Not mentioned	ELISA	BK, BK 1–8
Gangnus et al. [31]	28	-	26.5	-	-	-	-	3 mg/mL HDMB, 10.8 µg/mL nafamostat, 31 mg/mL EDTA, 5.9 mg/mL citrate, 1% formic acid, 409 ng/mL omapatrilat, and 515 µg/mL chloroquine	LC-MS/MS	BK, BK 1–8, KD, KD 1–8
Hilgenfeldt et al. [32]	10	-	32.8 ± 2.2/ 27.8 ± 4.0	-	CBP	116.8 ± 6.1	67.8 ± 6.7	2 ml; 10 μL o-phenanthroline, 16.7 μL polypropylene, 0.0625% EDTA	Amidolysis	Ang I, BK, KD
Lindstrom et al. [33]	19	3/16	48.0	-	-	-		Aprotinin (10,000 KIU/ml), trypsin inhibitor (800 µg/L), HDMB (4 mg/ml), 1,10-phenanthrolin (10 mg/ml) and EDTA-K2 (20 mg/ml)	LC-MS/MS	BK
Moraes et al. [24]	8		38.0 ± 3.9		CBP	109.0 ± 4.0	83.0 ± 1.7	Not mentioned		BK, BK 1–8
Murphey et al. [50]	12	6/6	25.0 – 49.0^	-	-	-	-	Not mentioned	LC-MS/MS	BK 1–5
Seip et al. [52]	6	-	-	-	-	-	-	Not mentioned	Ion trap MS	BK 1–5
Van den Broek et al. [34]	6	-	-	-	-	-	-	Not mentioned	HPLC	BK, BK 1–8
Mkhize et al. [47]	40	18/22	37.0 ± 6.3	-	-	-	-	Not mentioned	ELISA	Ang II, Ang 1–7
Basu et al. [25]	36	22/14	52.5	-	CBP	-	-	EDTA, 1,10-phenanthroline, pepstatin A, PHMB, AEBSF, and specific inhibitors for renin and aminopeptidases A and N	LC-MS/MS	Ang I, Ang II, Ang III, Ang IV, Ang 1–5, Ang 1–7, Ang 1–9, Ang 2–7, Ang 3–7, Ang 2–10

*AESBF* - 4-(2-Aminoethyl)benzene sulfonyl fluoride hydrochloride, *ABPM* Ambulatory Blood Pressure Monitoring (24 hr average of SBP ≥ 130 and/or DBP ≥ 80 mmHg for hypertension), *CBP* Clinical Blood Pressure (SBP ≥ 140 and/or DBP ≥ 90 mmHg for hypertension), *DBP* Diastolic Blood Pressure, *EDTA* Ethylenediaminetetraacetic acid, *HDMB* Hexadimethrine Bromide, *PCMB* p-Chloromercuribenzoic acid, *PHMB* p-hydroxymercuribenzoic acid, *SBP* Systolic Blood Pressure.

**‘'#'** – Unless stated, value for total number of participants, **‘**-‘– Not Reported, **‘^’** – Only age range reported.

**Table 2:** Study Characteristics for Hypertensive Studies.

Author	Sample Size	Gender (M/F)^#^	Age (yr) (Mean ± S.D)^#^	Ethnicity	Index Test	BP Readings		Protease Inhibitor Used	Sample Analysis Methods	Peptides Measured
						SBP (Mean ± S.D)^#^	DBP (Mean ± S.D)^#^			
Campbell *et al*. [40]	19	9/10	56		CBP	-	**-**	2 _mol CGP 38560 A (renin inhibitor), 50 mmol/L 1,10-phenanthroline, 1 g/L neomycin sulfate, 125 mmol/L EDTA, and 2% ethanol in water for RAS peptides, 4 mol/L guanidine thiocyanate for BK peptides and 1 mol/L HCL for kallidin peptides	Radioimmunoassay and ELISA	Ang I, Ang II, Ang III, Ang IV, Ang 1–7, Ang 1–9, Ang 1–12, BK, BK 1–7, BK 1–8, KD, KD 1–8
Ferrario *et al*. [49]	19	6/13	62.5 ± 8.5	-	CBP	131.0 ± 16.0	136.0 ± 13.0	1,10-ortho-phenanthroline monohydrate (0.5 mmol/L); PHMB	Radioimmunoassay	Ang 1–12
Moraes *et al*. [24]	10	10	44 ± 2.5	-	CBP	139.0 ± 3.8	102.0 ± 4.2	-	HPLC	BK, BK 1–8
Murphey *et al*. [50]	12	3	25 – 49^	-	-	-		Not mentioned	LC-MS/MS	BK 1–5
Silva *et al*. [39]	7	3/3	76.7 ± 6.6	-	-	-		0.44 mmol/ L o-phenanthroline, 1 mmol/L Na^+^, PCMB and 25 mmol/L EDTA	Radioimmunoassay	Ang II, Ang 1–7

*AESBF***-** 4-(2-Aminoethyl)benzene sulfonyl fluoride hydrochloride, *ABPM* Ambulatory Blood Pressure Monitoring (24 hr average of SBP ≥ 130 and/or DBP ≥ 80 mmHg for hypertension), *CBP* Clinical Blood Pressure (SBP ≥ 140 and/or DBP ≥ 90 mmHg for hypertension), *DBP* Diastolic Blood Pressure, *EDTA* Ethylenediaminetetraacetic acid, *HCl* Hydrochloric acid, *HDMB* Hexadimethrine Bromide, PCMB p-Chloromercuribenzoic acid, *PHMB* p-hydroxymercuribenzoic acid, *SBP* Systolic Blood Pressure.

**‘'#' –** Unless stated, value for total number of participants, **'**-'– Not Reported**, '^' –** Only age range reported.

### Data quality assessment

**Fig. 2:**
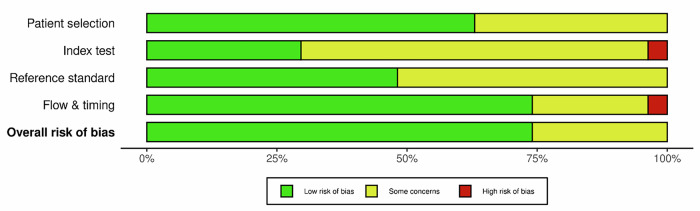
Risk of bias and applicability concerns graph: review authors’ judgements about each domain presented as percentages across included studies.

**Fig. 3:**
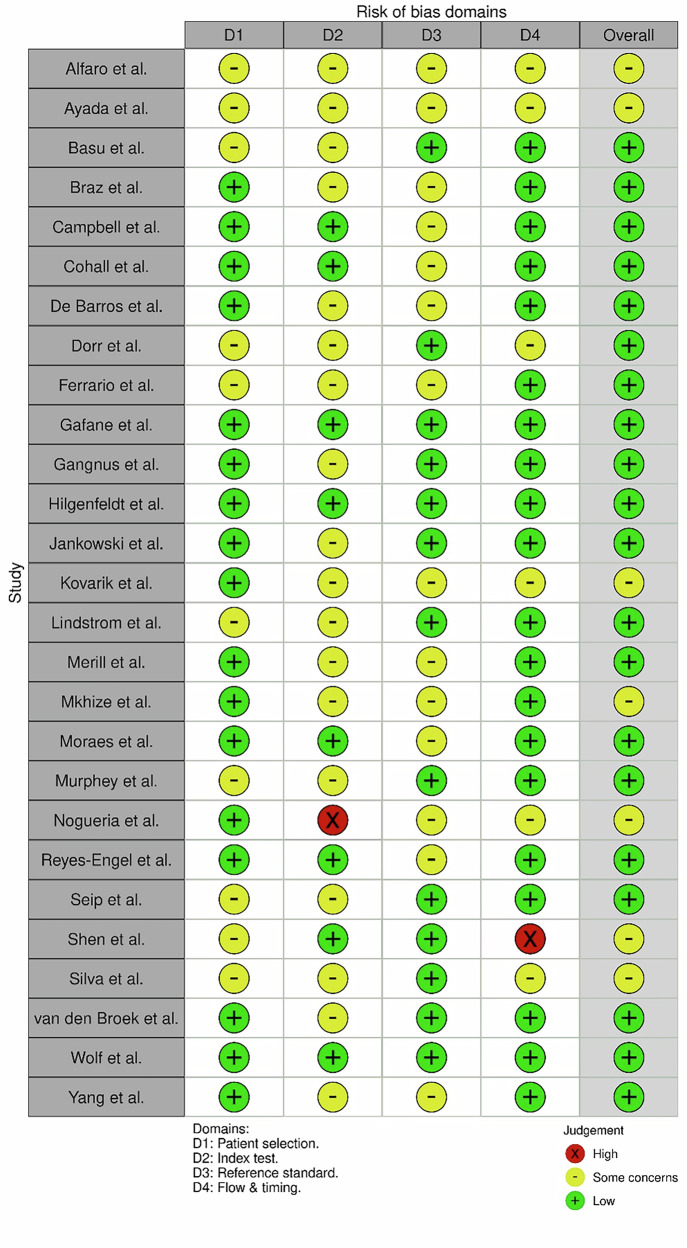
Risk of bias and applicability concerns summary: review authors’ judgements about each domain for each included study.

The Quality of Diagnostic Assessment Studies 2 (QUADAS-2) tool was used to assess all included studies [21]. This assessment analysed conduct of each study, its risk of bias, and the applicability of the study to the review’s aim using four parameters. The parameters were: 1) participant selection to investigate the inclusion and exclusion criteria and whether recruitment methods would introduce bias, 2) index tests such as BP measurement and pathology analysis for participant inclusion in the study and interpretation of results, 3) reference standard for measuring RAS or KKS peptide and whether they were interpreted independently and appropriately and 4) flow and timing to investigate if all participants received the same reference standard and if there was a delay between participant recruitment and sample analysis. The assessment outcomes were classified as low, high, and unclear risk of bias for the study (Figs. 2 and 3).

### Data syntheses

Due to the different units of measurement reported by the included studies on peptide levels, all the units were converted into fmol/mL. The Bland-Altman and Hozo model, as revised by Wan et al. [22], was used to derive the mean and standard deviation from studies that reported only median, quartiles, range (minimum and maximum), and sample size. The conversion and derivation equations for both analyses are provided in the Supplementary Material.

The parameters analysed were socio-demographics and postural position of participants during sample collection, inclusion of protease inhibitors, sample analysis methods, peptide measured and comparison of values in normotensive vs. hypertensive participants.

A quantitative meta-analysis was initially planned. However, due to substantial heterogeneity in the parameters stated above, the limited number of studies available on each peptide, and inconsistencies in data reporting, pooled meta-analysis was not feasible. Therefore, a narrative synthesis was performed, with only limited exploratory cross-study comparisons where sufficient data were available using RStudio (Version 2023.03.0).

## Results

### Study selection

**Fig. 4:**
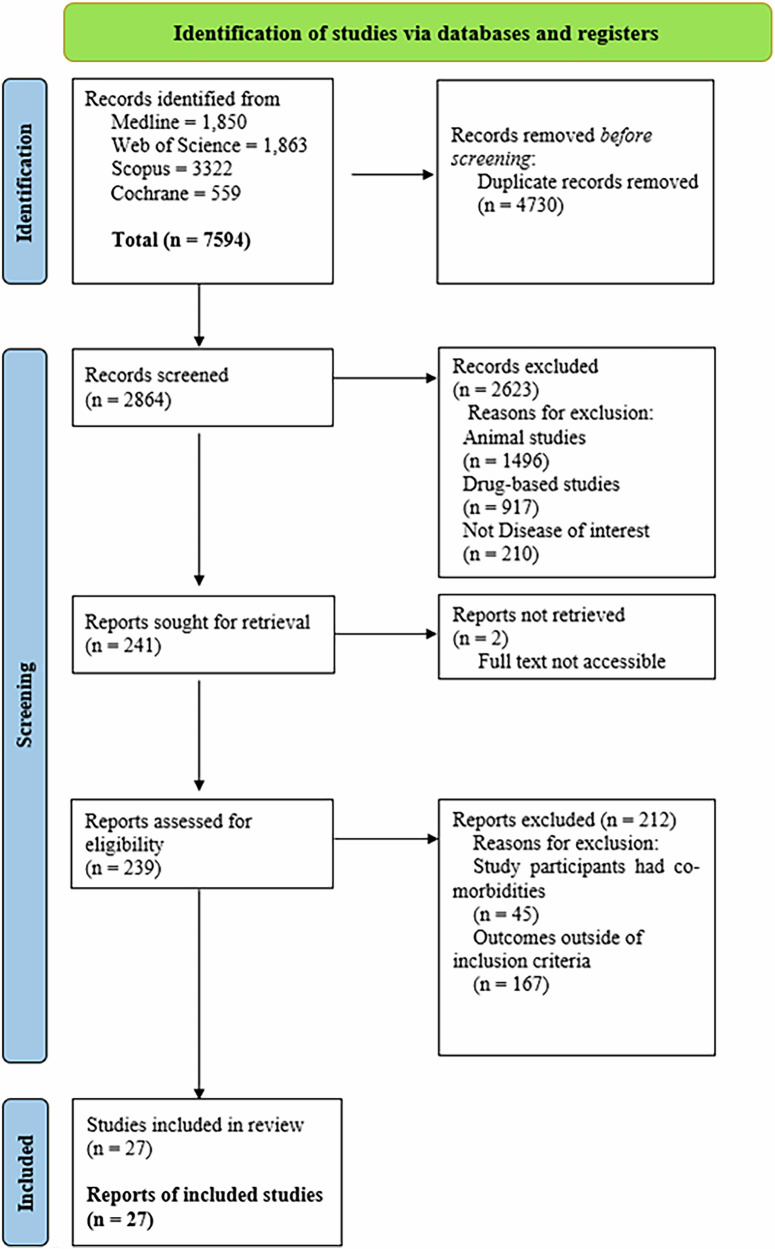
PRISMA Flowchart for Systematic Review Papers on the Renin-Angiotensin and Kallikrein-Kinin Systems.

The search generated 2864 articles, with 250 potentially eligible articles identified during the title and abstract screening. The full-text screen identified 27 articles that met the inclusion criteria of the review. The main reasons for exclusion were study design not meeting the inclusion criteria (69.9%), outcomes outside of the inclusion criteria (19.2%), and full text not available (0.8%) (Fig. 4).

### Participants characteristics

#### Studies with normotensive participants (Table 1)

For normotensive participants, there were twenty-six studies with a total number of 1758 participants and sample size ranging from 6–1116 and an average of 68 participants per study.

Seventeen studies reported sex, of which 49.6% were females (*n* = 873). Sixteen studies reported total mean age of 42.7 ± 12.6 years. Three studies reported the age of females separately with a mean of 32.9 ± 11.7 years, and two studies reported the age of males separately with a mean age of 28.6 ± 5.8 years.

Ethnicity was reported in only two of the studies as Afro-Caribbean, or Black (48.65%) and White South African (51.34%).

BP reporting was incomplete and limited. Eight studies measured mean baseline SBP of 120.4 ± 2.4 mmHg, and mean baseline DBP of 78.2 ± 1.9 mmHg. The remaining eighteen studies described participants as normotensive or healthy controls without providing BP readings.

Due to the inconsistent reporting of BP, age, sex, and ethnicity across normotensive studies, analyses involving these variables were limited.

#### Studies with untreated hypertensive participants (Table 2)

Five studies included untreated hypertensive participants, with a total of 67 participants and sample sizes ranging from 7–19, with a mean of 13 participants per study. Three studies reported sex, of which 57.7% were females (*n* = 26/45). Four studies reported age with a total mean age of 61.1 ± 16.4 years.

No study reported ethnicity.

BP data were also incompletely reported in hypertensive cohorts. Three studies measured SBP and DBP, but only two reported SBP readings, with a mean SBP of 135.3 ± 4.0 mmHg, and only one study reported DBP, with a mean DBP of 102 ± 4.2 mmHg.

The limited and inconsistent reporting of clinical characteristics, particularly BP, restricted robust comparisons between normotensive and hypertensive participants.

### Postural position

Reporting of participant posture during blood collection was limited. Among normotensive studies, only five explicitly stated that samples were collected in supine position [23–27], and these studies measured Ang I and Ang II. The remaining studies did not report posture, and combined with the difference in study characteristics, the available evidence did not permit inferences on effect of posture on peptide levels.

### Sample type

In normotensive studies, RAS peptides were measured in both serum and plasma. In serum, three studies measured Ang I and Ang II [28–30], reporting mean concentrations of 172 ± 189 fmol/mL and 68 ± 11 fmol/mL respectively [28–30]. Two studies measured Ang 1–7 in serum and reported a concentration of 2188.95 ± 2985.54 fmol/mL [29, 30].

The remaining studies measured the peptides in plasma with mean concentrations of 100 ± 111 fmol/mL for Ang I, 1976.59 ± 6032.82 fmol/mL for Ang II, and 87.04 ± 137.51 fmol/mL for Ang 1–7.

Although there was no statistically significant difference between serum- and plasma-based studies, interpretation is limited by the small number of serum-based studies and heterogeneity in study characteristics.

For KKS peptides, six studies measured BK in plasma and reported a mean concentration of 380 ± 530 fmol/mL [7, 24, 30–33], whereas one study measured BK in serum and reported substantially higher concentrations of 85.5 × 10^3^ ± 37.44 × 10^3^ fmol/mL [34]. Similarly, Bradykinin 1–8 (BK 1–8) was reported at 2410 ± 3420 fmol/mL in plasma [7, 24] and 81.85 × 10^3^ ± 42.88 × 10^3^ fmol/mL in serum [34].

Although these findings suggest that there may be variation of peptide levels in different matrices, firm inference was limited by the small number of serum-based studies.

### Protease inhibition

#### Studies with normotensive participants (Table 1)

Across the twenty-six normotensive studies, twelve (46%) reported the use of protease inhibitors at time of sample collection. The inhibitor combinations varied between studies. Eight studies used combinations including o-phenanthroline, pepstatin A, p-Hydroxymercuribenzoic (PHMB), p-Chloromercuribenzoic acid (PCMB), 4-(2-Aminoethyl) benzene sulfonyl fluoride hydrochloride (AESBF), and Ethylenediaminetetraacetic acid (EDTA) [23, 26, 32, 35–39]. One study used AESBF, apronitin, bestatin, E-64, leupeptin, and EDTA [27]. One study used aprotinin, trypsin inhibitor, HDMB, o-phenanthroline, and EDTA [33]. One study used HDMB, nafamostat, citrate, formic acid, omapatrilat, chloroquine, and EDTA [31]. One study used ortho-phenanthroline monohydrate and PHMB [40].

#### Studies with untreated hypertensive participants (Table 2)

Among the five hypertensive studies, three (60%) reported the use of protease inhibitors at time of sample collection. Reported protease inhibitors included o-phenanthroline and PHMB [39], o-phenanthroline, PCMB, and EDTA [35], and CGP (renin inhibitor), phenanthroline, neomycin sulfate, ethanol in water (for RAS peptides), guanidine thiocyanate (for BK peptides), and HCl (for kallidin peptides) [41].

Overall, substantial heterogeneity was observed in use of protease inhibitors across included hypertensive studies.

### Methods used for sample analysis

Analytical methods also varied across all included studies. Twelve studies used immunoassay-based methods, enzyme-linked immunosorbent assay (ELISA), radioimmunoassay (RIA), or amidolysis [7, 26, 29, 32, 36–49].

Ten studies used LC-MS/MS [23, 25, 27, 28, 30, 31, 33, 34, 50, 51].

One study used liquid chromatography coupled with ion-trap mass spectrometry (LC/IT-MS) [52], one with high-performance liquid chromatography (HPLC) [24], and one with matrix-assisted laser desorption ionisation-time of flight mass spectrometry (MALDI-TOF MS) [53].

In exploratory comparisons of RAS peptides in normotensive studies, no statistically significant differences were observed between immunoassay-based studies and MS-based studies [Ang I; (120 ± 110 fmol/mL vs. 130 ± 180 fmol/mL), Ang II; (2170 ± 6350 fmol/mL vs. 110 ± 30 fmol/mL), Ang 1–7; (3270 ± 7410 fmol/mL vs 40 ± 50 fmol/mL)].

Similarly, no statistically significant differences were observed in measurement of KKS peptides in normotensive studies [BK; (50 ± 60 vs 21980 ± 42540 fmol/mL), BK 1–8; (4850 ± 2910 vs 40.84 × 10^3^ ± 57.71 × 10^3^ fmol/mL)].

However, these comparisons were based on small number of studies for each peptide, precluding any meaningful evaluation of influence of analytical methods on peptide levels.

### Peptide levels

#### Studies with normotensive participants (Table 3)

Across normotensive studies, reported peptide concentrations spanned a wide range such as 1680 ± 5499 fmol/mL for Ang II, 500 ± 1070 fmol/mL for Ang 1–7, 10 ± 10 fmol/mL for Angiotensin A (Ang A), and BK being 12580 ± 32290 fmol/mL. No study measured Alamandine and Bradykinin 1–7 (BK 1–7).

**Table 3:** Peptide Levels in Normotensive and Hypertensive Participants.

Peptide	Role	Concentration (Normotensive)	Concentration (Hypertensive)
		Mean ± S.D (fmol/mL)	Mean ± S.D (fmol/mL)
Ang I	Precursor to angiotensin peptides [5]	124 ± 134	1.2 ± 0.83
Ang II	Binds to AT_1_R to induce vasoconstriction [5]	1680 ± 5499	20 ± 30
Ang III	Binds to AT_1_R to induce vasoconstriction and AT_2_R to induce vasodilation [8]	10 ± 10	0.093 ± 0.055
Ang IV	Binds to AT_4_R to induce vasoconstriction and AT_2_R to induce vasodilation [8]	20 ± 10	0.15 ± 0.089
Ang 1–5	Binds to AT_2_R to induce vasodilation [5]	30 ± 30	-
Ang 1–7	Binds to AT_2_R/MasR to induce vasodilation [5]	500 ± 1070	200 ± 280
Ang 1–9	Binds to AT_2_R to induce vasodilation [8]	20	0.061 ± 0.0384
Ang 1–12	Precursor to angiotensin peptides and binds to AT_1_R to induce vasoconstriction [56]	1250	1460 ± 350
Alamandine	Binds to MRGD to induce vasodilation [5]	-	-
Ang A	Binds to AT_1_R to induce vasoconstriction and binds to AT_2_R to induce vasodilation [5]	10 ± 10	-
Bradykinin	Binds to B_2_R to induce vasodilation [28]	380 ± 530 and (85.8 ± 37.44) × 10^3x^	20 ± 30
Bradykinin 1–5	Binds to B_2_R to induce vasodilation [47]	340 ± 100	20 ± 3.0
Bradykinin 1–7	Binds to B_2_R to induce vasodilation [57]	-	0.426 ± 0.2
Bradykinin 1–8	Binds to B_2_R to induce vasodilation [28]	2410 ± 3420 and (81.85 ± 42.88) × 10^3x^	0.02 ± 0.03
Kallidin	Binds to B_2_R to induce vasodilation [28]	50 ± 100	0.20 ± 0.15
Kallidin 1–8	Binds to B_2_R to induce vasodilation [28]	0.2 – 2.0	0.0974 ± 0.074

-=no study found on levels of peptides.

x=higher levels due to ex vivo formation of peptides.

Because only 8 out of 26 normotensive studies reported BP data, assessment of relationships between peptide levels and BP was limited. Exploratory analyses of Ang I and Ang II did not identify statistically significant associations with SBP and DBP. However, these analyses were underpowered and could not be inferred as evidence of no relationship.

Sex- and ethnicity-specific reporting was also limited. Across included studies, there was no sex-based differences for Ang I, Ang II, or Ang 1–7 in pooled descriptive comparisons. However, study-specific analyses showed that in two individual studies, Ang 1–7 was significantly higher in women [26, 38].

Study-specific analysis in another individual study also revealed ethnicity-specific significant differences with higher Ang I and Ang II concentration in young White adults compared to young Black adults [28].

No ethnicity data were reported in studies measuring KKS peptides.

#### Studies with hypertensive participants not on treatment (Table 3)

Across hypertensive studies, peptide concentrations were generally lower than those reported in normotensive studies with Ang II as 20 ± 30 fmol/mL, Ang 1–7 as 200 ± 280 fmol/mL, and BK as 20 ± 30 fmol/mL. No study measured Angiotensin 1–5 (Ang 1–5), Alamandine, Ang A, and Bradykinin 2–9 (BK 2–9).

Some peptides such as Ang 1–5, Alamandine, or Ang A were also not measured in hypertensive cohorts. One RCT that included a placebo arm reported very low concentrations of RAS and KKS peptides in participants allocated to placebo arm [41].

Overall, these observations were based on few studies with incomplete reporting of BP and participant characteristics, and methodological heterogeneity. As such, interpretation was limited to descriptive findings rather than as evidence of a specific biological mechanism.

## Discussion

This systematic review synthesised reported baseline circulating concentrations of RAS and KKS peptides in normotensive and hypertensive adult populations. To the best of our knowledge, this is the first review to evaluate both reported peptide concentrations and methodological factors that influence their measurement in these two populations.

### Participant characteristics

Age, sex, and ethnicity impact RAS and KKS peptide concentrations [54]; however, these associations were not explored in most of the included studies. Furthermore, BP data were also incompletely reported. As a result, it was not possible to robustly evaluate the extent to which participant characteristics contributed to differences in peptide levels across studies.

Some individual studies suggested possible statistically significant differences based on sex and ethnicity. For example, higher Ang 1–7 levels were reported in women in normotensive cohorts [26, 38], suggesting a possible upregulation of the non-canonical RAS axis in women. Higher Ang II levels in a young White population together with the inverse relationship in a young Black population by another study also indicates a possible upregulation of canonical RAS based on ethnicity [28].

Overall, the current literature is insufficient to define peptide reference levels according to age, sex, or ethnicity. Future studies should incorporate more detailed and consistent participant characteristics to evaluate their clinical applicability.

These findings also highlight research gaps that could be explored and may inform personalised therapeutic strategies investigating the influence of age, sex, and ethnicity on peptide levels.

The relationship between BP and peptide levels was also difficult to assess. Only a minority of studies reported SBP and DBP values and cross-study comparisons were limited and non-significant. Given the sparse and inconsistent reporting, these analyses were exploratory only. However, as the RAS and KKS are BP control and maintenance systems, this is an important limitation which needs to be addressed in future studies.

### Postural Position

Variability in sample collection methods affects peptide levels [16]. Postural position, for example, influences RAS and KKS peptides [17]. In an upright position, blood flow shifts from central to peripheral veins, decreasing right atrial pressure and activating BP pathways. In a supine position, blood flow equilibrates, accurately reflecting circulating peptide levels [17]. However, reporting of posture was poor in the included studies. Only five normotensive studies explicitly reported supine sample collection, while most studies did not report participant posture at all.

Because studies also differed in their characteristics, the available evidence does not allow robust conclusions on effect of posture on peptide levels. Rather than suggesting that posture is unimportant, these findings highlight incomplete methodological reporting and the need for standardised sampling conditions in future studies.

### Protease inhibition

Similarly, the use of protease inhibitors at time of sample collection varied, with different protease inhibitor cocktails being used. This is an important source of methodological variability, given the short half-life of several RAS and KKS peptides and the potential for ex vivo degradation and peptide interconversion during sample handling [6, 55].

Furthermore, many of the reported inhibitor cocktails were broad or non-standardised, and not all were clearly designed to comprehensively inhibit metal-dependent proteases and amino- and carboxy- peptidases relevant to both RAS and KKS [55].

This raises the possibility of incomplete peptide stabilisation across studies. This limits the comparability of reported levels and is likely to have contributed to between-study variation. Future research is needed to develop and validate a protease inhibitor cocktail targeting all enzymes in both RAS and KKS for reliable and accurate circulating peptide levels.

### Methods of peptide analysis

Peptide analysis methods also varied. This variability is important because the RAS and KKS peptides are structurally similar, and analytical specificity is critical for accurate quantification [8]. MS is considered the gold standard due to its high sensitivity and specificity [56], while immunoassays have limitations such as cross-reactivity and high false positives [18].

Although exploratory comparisons in this review did not show statistically significant differences between immunoassay-based and MS-based studies for several peptides, these analyses were based on small numbers of studies and were confounded by broader methodological heterogeneity.

Therefore, the present findings should not be interpreted as evidence that analysis methods are equivalent. Rather, they reinforce the need for standardised and analytically robust approaches, particularly if clinically meaningful reference ranges of RAS and KKS peptides are to be established.

### Peptide levels

Overall, research on the non-canonical arm of the RAS and the KKS was less extensive than that on the canonical RAS, with the majority of studies undertaken in normotensive populations. Reported peptide concentrations varied widely from 0.06–81.85 × 10^3^ fmol/mL, and this broad range likely reflects both biological variation and methodological heterogeneity.

Across all included studies, there were lower levels of several circulating RAS and KKS peptides in hypertensive cohorts than in normotensive cohorts. However, this finding should be interpreted cautiously as indicated earlier, there was a small number of hypertensive studies, BP was incompletely reported, and there was substantial methodological heterogeneity in each study design.

Thus, this review does not indicate mechanistic conclusions such as depletion or suppression of canonical or non-canonical pathways in hypertension. Instead, it shows that reported levels vary across different studies, and the available evidence is insufficient to determine whether observed differences are biological, methodological, or both.

Angiotensin 1–12 (Ang 1–12) was the only peptide with relatively higher circulating levels in hypertensive cohorts. Due to its role as a substrate for chymase-mediated formation of Ang II in somatic tissues [57, 58], it could be indicative of activation of canonical RAS in tissues, which may not be detected by using plasma or serum-based assays and could be a potential research avenue for investigating differences in tissue RAS from systemic RAS.

A further gap in literature is the incomplete measurement of several peptides across cohorts. Ang 1–5 and Ang A were not measured in hypertensive studies, BK 1–7 was not measured in normotensive studies, and Alamandine was not measured in either group. These prevented a comprehensive comparison of all RAS and KKS peptides and further limited assessment of whether a broader circulating peptide profile could improve understanding of normotension and hypertension.

### Strengths and limitations

A strength of this review is that it evaluates both RAS and KKS peptide measurements and examines pre-analytical and analytical factors that may influence reported levels. However, several limitations should be acknowledged. First, the included studies were heterogeneous in sample collection and peptide stabilisation strategies, analysis methods, and reported levels. Second, BP readings were incompletely reported, limiting clinically relevant comparisons. Third, several peptides were measured in only a small number of studies or not measured at all in one or both cohorts. Fourth, the exclusion of grey literature, including conference abstracts, may have resulted in omission of relevant data, although this decision was made because such sources typically lack the detailed methodological reporting required for this review. Fifth, full-text screening was conducted by a single reviewer, which may have introduced selection bias.

Taken together, these limitations indicate that the current evidence is insufficient to establish reference ranges for circulating RAS and KKS peptides or to define their diagnostic or therapeutic potential in hypertension.

Larger studies with standardised protocols and measurement of all RAS and KKS peptides are needed before these peptides can be reliably interpreted in clinical or translational settings.

## Conclusion

This systematic review shows that reported circulating levels of RAS and KKS peptides vary widely across studies of normotensive and hypertensive adults, and that heterogeneity exists in sample collection and peptide stabilisation, reported clinical phenotypes, and analytical methods. Although some peptides were reported at lower levels in hypertensive cohorts than in normotensive cohorts, the heterogeneity in the current evidence currently limits interpretation and is insufficient to establish reference ranges of the peptides.

Standardised pre-analytical handling, comprehensive protease inhibition, consistent reporting of BP and clinical phenotypes in large, well-characterised cohorts together with analytically robust platforms such as MS will be essential for future studies to clinically define their reference ranges.

This may ultimately contribute to hypertension stratification, treatment selection, and development of novel therapeutic measures for treating hypertension.

## Summary

### What is known about the topic

- Hypertension is the leading global risk factor for cardiovascular diseases (CVDs).
- The renin-angiotensin system (RAS) and kallikrein-kinin system (KKS) are central to BP regulation and involve complex interactions of vasoactive peptides.
- The reference ranges for RAS and KKS peptides in normotensive and hypertensive populations remain poorly established, limiting their clinical utility in diagnosis and treatment.

### What this study adds

- This systematic review is the first to synthesise reported baseline circulating concentrations of both RAS and KKS peptides in normotensive and hypertensive adults alongside the methods used to measure them.
- The review demonstrates substantial heterogeneity in use of protease inhibitors at time of sample collection for peptide stability, reporting of BP values, participant characteristics, and absence of data for several peptides in one or both normotensive and hypertensive cohorts.
- Future studies need to incorporate robust and standardised methodologies and leverage advanced analytical techniques such as LC-MS/MS with the aim of establishing clinically relevant reference ranges, stratified by age, sex, and ethnicity, for diagnosis and treatment in precision medicine.

## Supplementary information

Supplemental Material

## Data Availability

All data generated or analysed during this review are included in this published article.
