# Supplementary material for: A systematic review of the measurement of the renin-angiotensin and kallikrein-kinin system peptides in normotension and hypertension
Source: J Hum Hypertens. 2026 Jul 30;40(8):583–95. doi: 10.1038/s41371-026-01186-x (PMC13457092; doi:10.1038/s41371-026-01186-x)
Supplement: Supplementary file 1 [file 41371_2026_1186_MOESM1_ESM.docx]

**Search Strategy**

1.	(Ang I or Ang II or Ang III or Ang 1-7 or Ang 1-12 or Ang 1-9 or Alamandine or Ang 1-5 or Ang IV or Ang A or MasR or MRGD or ACE2 or ACE2a or ACE2b).mp.

2.	(Angiotensin I or Angiotensin II or Angiotensin III or Angiotensin IV or Angiotensin 1-5 or Angiotensin 1-7 or Angiotensin 1-9 or Angiotensin 1-12 or Alamandine or Angiotensin A or ACE2 or ACE2a or ACE2b or MRGD or MASR).mp.

3.	(bradykinin or kallidin or lys-bradykinin or Des-Arg9-BK or BK 1-9 or BK 1-5 or BK 1-7 or BK 1-8 or BK 1-3 or kinin*).mp. [mp=title, book title, abstract, original title, name of substance word, subject heading word, floating sub-heading word, keyword heading word, organism supplementary concept word, protocol supplementary concept word, rare disease supplementary concept word, unique identifier, synonyms]

4.	1 or 2 or 3

5.	renin-angiotensin system.mp. or Renin-Angiotensin System/

6.	renin-angiotensin-aldosterone system.mp.

7.	kallikrein-kinin system.mp. or Kallikrein-Kinin System/

8.	4 or 5 or 6 or 7

9.	(blood or plasma or serum).mp.

10.	(level* or concentration* or measure*).mp.

11.	8 and 9

12.	Hypertension/ or hypertension.mp.

13.	normotension or healthy or health*.mp.

14.	essential hypertension.mp. or Essential Hypertension/

15.	hypertensi*.mp.

16.	(high adj2 blood pressure).mp.

17.	12 or 13 or 14 or 15 or 16

18.	4 and 8 and 11 and 17

19.	exp animals/ not humans.sh.

20.	18 not 19

**Conversion of peptide levels to fmol/mL**

**pg/mL to fmol/mL**

$$fmol/mL =\frac{peptide concentration\left( pg/mL \right)\times1000}{MW \left( g/mol \right) of peptide}$$

**ng/mL to fmol/mL**

$$fmol/mL =\frac{peptide concentration\left( ng/mL \right)\times{10}^{6}}{MW \left( g/mol \right) of peptide}$$

**Calculation of sample mean** $\left( \overline{X} \right)$ **using the lower boundary, (LB), upper boundary (UB), minimum value (a), maximum value (b), sample size (n)**

$\overline{X} =\frac{LB_{1}+ UB_{1}}{2}+\frac{a + 2m + b}{4}+\frac{a - 2m + b}{4n}$

**Calculation of standard deviation (S) using the minimum value (a), maximum value (b)**

$$S \approx\frac{b - a}{\sqrt{12}}$$
